# Phylogeny and androgenesis in the invasive *Corbicula *clams (*Bivalvia*, *Corbiculidae*) in Western Europe

**DOI:** 10.1186/1471-2148-11-147

**Published:** 2011-05-27

**Authors:** Lise-Marie Pigneur, Jonathan Marescaux, Kathleen Roland, Emilie Etoundi, Jean-Pierre Descy, Karine Van Doninck

**Affiliations:** 1 Research Unit in Environmental and Evolutionary Biology (URBE), University of Namur (FUNDP), Rue de Bruxelles, 61, 5000 Namur - Belgium

## Abstract

**Background:**

The genus *Corbicula *is one of the most invasive groups of molluscs. It includes both sexual and androgenetic lineages. The present study re-assessed the different morphotypes and haplotypes of West European *Corbicula *in order to clarify their taxonomic identification and phylogenetic relationships with American and Asian *Corbicula *clams. We studied several populations from West European river basins (Meuse, Seine, Rhine and Rhône) through an "integrative taxonomy" approach. We combined morphology, partial mitochondrial *COI *and *cyt b *sequences and eleven microsatellite loci. Furthermore, we looked for discrepancies between mtDNA and nrDNA/morphology, indicative of androgenesis between lineages.

**Results:**

There are three *Corbicula *morphotypes in Western Europe associated to three mitochondrial lineages and three genotypes. Form R shares the same *COI *haplotype as the American form A and the Japanese *C. leana*. Form S and the American form C have the same haplotype, although their morphologies seem divergent. The European form Rlc belongs to the same mitochondrial lineage as both the American form B and the Asian *C. fluminea*.

Interestingly, within each haplotype/genotype or lineage, no genetic diversity was found although their invasive success is high. Moreover, we detected rare mismatches between mtDNA and nrDNA/morphology, indicative of androgenesis and mitochondrial capture between form R and form S and therefore challenging the phylogenetic relatedness and the species status within this genus. The global phylogenetic analysis revealed that the sexual *Corbicula *lineages seem restricted to the native areas while their androgenetic relatives are widespread and highly invasive.

**Conclusions:**

We clarified the discrepancies and incongruent results found in the literature about the European morphotypes of *Corbicula *and associated mitochondrial lineages. The three West European morphotypes belong to three distinct nuclear and mitochondrial lineages. However mitochondrial capture occurs in sympatric populations of forms R and S. The species status of the morphotypes therefore remains doubtful. Moreover the androgenetic lineages seem widely distributed compared to their sexual relatives, suggesting that androgenesis and invasive success may be linked in the genus *Corbicula*.

## Background

The clams of the genus *Corbicula *are successful fresh and brackish water invaders considered 'r'-strategists, with rapid maturation, high fecundity, and high dispersal [1-3]. These bivalves are benthic filter-feeders which can reduce phytoplankton density [4-6], compete with native species [7,8] and damage industrial cooling systems [9]. The genus *Corbicula *is of particular interest both because of its diverse reproductive strategies (from free-swimming larvae to incubation of larvae in gills) [10] and because it contains sexual and asexual reproducing lineages. The genus includes sexual dioecious species as well as hermaphrodites, with at least some or all of the latter reproducing through a rare form of asexual reproduction, known as androgenesis, in which offspring are clones of their father [11,12]. Androgenesis in *Corbicula *is characterized by the fertilization of an oocyte by an unreduced sperm (with a DNA content equal to the DNA content of a somatic cell). The maternal nuclear DNA is then entirely extruded as two polar bodies and only the 'male' pronucleus remains and becomes the nucleus of the zygote [11-14].

Interestingly, androgenetic *Corbicula *lineages have biflagellate sperm while sexual ones are characterized by monoflagellate sperm [15-19].

The modern native range of the genus *Corbicula *is Asia, the Middle East, Australia and Africa but fossils have been recorded in Europe, North America and Japan (reviewed in [20]). The first record of *Corbicula *outside its extant original range was in 1924 in British Columbia [21,22]. The clams then quickly spread throughout North America and arrived in South America in the 1970s [23] and in Europe in the 1980s [24].

Although *Corbicula *is one of the most important invasive bivalve groups because of its ecological and economic impacts, the taxonomic status of the invaders remains unresolved, because, amongst others, the genus shows considerable phenotypic variation in shell shape and ornamentation, some of which ecophenotypic [25]. The first traditional morphology-based taxonomic studies of Asian *Corbicula *described approximately 200 species [26-28]. However, subsequent studies based on shell characteristics [29], allozymes [25] or genetics [30] suggested the occurrence of fewer species. In addition, both dioecious sexual and hermaphroditic clonal lineages were found in Asia [17,31] but their evolutionary relationships, taxonomic status and nomenclature are still uncertain. Park & Kim [31] studied several Asian populations of *Corbicula *and showed that the estuarine species form a distinct sister-clade of the freshwater species with two mitochondrial lineages being the most common in freshwater habitats in Asia. Within these two major lineages, several haplotypes are also found in the invaded areas (America and Europe), e.g. haplotype FW1 in North America and haplotype FW5 in America and Europe [31] (Table 1).

**Table 1:** GenBank accession numbers, *COI *haplotype designation and localities of *Corbicula *spp. sequences included in phylogenetic analysis.

Code^1^	Taxon	Location	Haplotype	GenBank	Sperm morphology
**sandai A**	*C. sandai*	Japan		AF196272	monoflagellate³
**sandai B**	*C. sandai*	Japan		AF196273	monoflagellate³
**austr**	*C. australis*	Australia		AF196274	Biflagellate^4^
					
**FW1**	*C. fluminea*	Korea		AF196269	biflagellate³ ^α^
**FW1**	C. sp. (form B)	USA	B1-3	AF519509-11	biflagellate^5^
**C2**	C. sp. (form C) ^2^	Argentina		AF519512	
**FW2**	C. sp.	China		AF457989	
**FW3**	C. sp.	China		AF457990	
**FW4**	*C. fluminalis*	Netherlands	V	AF269096-8	
**FW4**	C. sp. (form Rlc)	*See *Table 4	3	GU721084	biflagellate^6^
**FW4**	*C. subplanata*	Indonesia		DQ285602	
**lindu**	*C. linduensis*	Indonesia		DQ285579	monoflagellate^7^
					
**FW5**	*C. fluminea*	France	I	AF269090-3	
**FW5**	C. sp. (form A)	USA	A1-13	AF519495-507	biflagellate^5^
**FW5**	C. sp. (form R)	*See *Tables 3, 4	1	GU721082	biflagellate^6^
**FW5**	*C. leana*	Japan		AF196268	biflagellate³ ^α^
**FW5**	C. sp.	Germany	H2	AY097263-75	
**FW7**	*C. fluminea*	France	II	AF269094	
**FW8**	C. sp.	Taiwan		AF457991	
**H7**	C. sp.	Germany		AY097284	
**FW9**	*C. javanica*	Indonesia		AF457993	
					
**FW10**	C. sp.	Korea		AF457992	
**FW11**	C. sp.	China		AF457994	
**FW12**	C. sp.	China		AF457995	
**FW13**	C. sp.	China		AF457999	
**FW14**	*C. fluminea*	Thailand		AF196270	
**FW15**	C. sp.	Vietnam		AF468017	
**FW16**	C. sp.	Vietnam		AF468018	
					
**FW17**	C. sp. (form S)	*See *Tables 3, 4	2	GU721083	biflagellate^6^
**FW17**	*C. fluminea*-like	France (Rhône)	IV	AF269095	
**FW17**	C. sp. (form C)	Argentina	C1	AF519508	biflagellate^5^
**FW17**	C. sp.	Germany	H4	AY097277-81	
**H1**	C. sp.	Germany		AY097262	
**H5**	C. sp.	Germany		AY097282	
**H8**	C. sp.	Germany		AY097285	
					
**H18**	C. sp.	Israel		AY097295-8	
**H25**	C. sp.	France (Rhône)		AY097302	
**H26**	C. sp.	France (Rhône)		AY097303	
**fluminalis A**	*C. fluminalis *A	China		AF457996	
**fluminalis C**	*C. fluminalis *C	China		AF457998	
**H32**	C. sp.	Japan		AY097312	
**japonica A**	*C. japonica*	Japan		AF196271	monoflagellate^7^
**Kor4**	C. sp.	Korea		EU090399	
**KR1**	*C. japonica*	Korea		AF367440	monoflagellate^7^
**lamar**	*C. lamarckiana*	Thaïland		DQ285578	monoflagellate^8^
**loeh80**	*C. loehensis*	Indonesia		DQ285580	monoflagellate^8^
**loeh81**	*C. loehensis*	Indonesia		DQ285581	monoflagellate^8^
**mata89**	*C. matannensis*	Indonesia		DQ285589	monoflagellate^8^
**mata63**	*C. matannensis*	Indonesia		AY275663	monoflagellate^8^
**mata65**	*C. matannensis*	Indonesia		AY275665	monoflagellate^8^
**mata92**	*C. matannensis*	Indonesia		DQ285592	monoflagellate^8^
**mata87**	*C. matannensis*	Indonesia		DQ295587	monoflagellate^8^
**mata94**	*C. matannensis*	Indonesia		DQ285594	monoflagellate^8^
**posso**	*C. possoensis*	Indonesia		DQ285598	monoflagellate^8^
**anomio**	*C. anomioides*	Indonesia		DQ285605	
**mada**	*C. madagascariensis*	Madagascar		AF196275	
**Neocorbicula limosa**	*N. limosa*	Argentina		AF196277	monoflagellate^8^

Sperm morphology is indicated if known: biflagellate sperm is considered as indicative for androgenesis [19], ^1^haplotype name used in the present study, ^2^mitochondrial-morphotype mismatch, ³ [15], ^4 ^[16], ^5 ^[18], ^6^present study, ^7 ^[41], ^8 ^[17], ^α^androgenetic forms as evidenced by cytological studies [11,12].

In the New World, three shell morphotypes have been distinguished for invasive *Corbicula *[18]. The two North American morphs show substantial morphological and genetic differences [32,33] with form A (also referred to as the "white form") being apparently derived from populations of *C. leana *from Japan and form B (the "purple form") being derived from populations of *C. fluminea *from China and/or Korea [34,19]. Both morphs have also been recorded in South America where a third, genetically distinct, morph also occurs (form C; [18]).

In Europe, *Corbicula *clams were first recorded in French and Portuguese estuaries in 1980 [24]. They have subsequently succeeded in colonising many of the major European watersheds and can reach high densities in some rivers and lakes ([35-39], authors' personal observation). Ever since *Corbicula *was found in Europe in the 1980s, it has been debated which species invaded this continent and where they came from. A study [40] revealed two morphotypes in several European rivers. They were identified as *C. fluminea *and *C. fluminalis. C. fluminalis *sensu stricto is a hermaphroditic form from Central Asia, the Caucasus and Middle East, characterized by a high triangular shell [41]. Both morphotypes found in European rivers were genetically characterized as two distinct genetic lineages based on allozymes and mitochondrial *COI *sequences. However, in the River Rhône, Renard *et al*. [40] found none of these two haplotypes but instead found a unique, third haplotype (*COI *haplotype IV) with the morphology of the "*C. fluminea*" lineage (Table 2). Based on enzyme polymorphism and RFLP analysis of the mitochondrial *COI *gene, this cryptic, unnamed lineage was considered a separate species ("*C. fluminea*-like") [40]. Pfenninger *et al*. [42] also conducted a genetic study on *Corbicula *including European samples. Two morphotypes, the "round form" (R) and the "saddle form" (S), were found sympatrically in the River Rhine. Based on mitochondrial *COI *sequence data, the round form (R) from the Rhine has the haplotype H2 which is identical to haplotype I of Renard *et al*. [40], American form A [18,19,34] and Asian *C. leana *haplotype FW5 [31,34]. However, form S from the Rhine, which is morphologically close to the *C. fluminalis *of Renard *et al*. [40], has exactly the same *COI *haplotype (haplotype H4) as the third genetic lineage (haplotype IV) from the Rhône and not the expected *C. fluminalis *haplotype. These *COI *data also indicate that the haplotype H4 of form S, and hence Renard *et al*.'s [40] haplotype IV, is identical to the haplotype C1 of American form C ([18,19]). Moreover, random DNA amplification fingerprinting and ITS1 RFLP analysis revealed the presence of cryptic hybrids between the R and S lineages from the River Rhine [42]. These hybrids could display either parental morphotypes or, in a few cases, intermediate morphotypes. In Portugal, the populations of *Corbicula *from the Lima and Minho rivers are morphologically distinct but have the same *COI *haplotype, identical to haplotype FW5 (the "form A - form R lineage") [43]. The observed difference in shell shape and colour has been attributed to an adaptation to distinct ecological conditions, different origins and/or genetic alterations during distinct migration, or differential selection processes in the two rivers [43].

**Table 2:** Comparison of the *COI *results for the different *Corbicula *morphotypes including the previous nomenclature used.

Renard *et al*. 2000 [40]	Haplotype I (FW5) *C. fluminea*	Haplotype IV (FW17) *C. fluminea*-like (Rhône)	Haplotype V (FW4) *C. fluminalis*
Pfenninger *et al*. 2002 [42]	Haplotype H2 (FW5) Form R		Haplotype H4 (FW17) Form S

**Present study**	**Haplotype 1 (FW5) Form R ** **Genotype 1 ** **Haplotype 2 (FW17) Form R **(mt/morphotype mismatch: 2 individuals) **Genotype 1**	**Haplotype 3 (FW4) Form Rlc (Rhône) Genotype 3**	**Haplotype 2 (FW17) Form S Genotype 2**

Asian haplotypes (Siripattrawan *et al*. 2000 [34]; Park & Kim 2003 [31])	*C. leana *Haplotype FW5	*C. fluminea *(FW1) *C. subplanata *Haplotype FW4	(Haplotype FW17)

American haplotypes (Lee *et al*. 2005 [18])	Haplotypes A1-13 (FW5) Form A	Haplotype B1-3 (FW1) Form B	Haplotype C1 (FW17) Form C

Representative *COI *haplotypes from each lineage have been included. See Table 1 and Fig. 2 for the whole data set. Genotypes (from microsatellite data) are indicated for each of the three forms investigated in the present study. Haplotype FW17 was not found in any Asian population but this nomenclature was used for the European haplotype IV [31].

Revising the divergent results obtained by Renard *et al*. [40] and Pfenninger *et al*. [42] for the "*C. fluminalis *- form S" morphotype (see also Table 2), it is still unclear which European morphotype of *Corbicula *corresponds to which haplotype and what is their phylogenetic relationship with American and Asian *Corbicula *clams. In Europe and America, most individuals of *Corbicula *that have the same morphotype also have the same mitochondrial haplotype. However a mismatch between mtDNA and morphotype may be observed because of androgenesis between distinct genetic lineages [18,19,44]. More specifically, an unreduced spermatozoon from one lineage can fertilize the egg of another lineage. The maternal nuclear DNA of the latter one will be extruded as two polar bodies but the mitochondria will be retained, while only the 'male' pronucleus of the first lineage will be involved in the zygote development [11,18,19]. This phenomenon has also been referred to as egg parasitism. In some rare cases, the maternal nuclear genome can be partially or totally retained, leading to a peculiar form of hybridisation [18].

Furthermore, Komaru *et al*. [45,46] observed in laboratory populations of *C. leana *and *C. fluminea *that incomplete extrusion of the maternal genome occurs, causing an elevation of ploidy level. Indeed, individuals of *C. fluminea *of several ploidy levels have been reported (2n: [47]; 3n: [48]; 4n: [48]).

The present study aimed at re-investigating the different morphotypes and haplotypes in West European lineages of *Corbicula *in order to finally clarify their phylogenetic relationships with American and Asian clams. We applied an "integrative taxonomy" approach by studying morphology and comparing it to molecular data [49]. More specifically, we used shell and sperm morphology, partial mitochondrial *COI *and *cyt b *sequences and, in addition, nuclear polymorphic microsatellite loci to study *Corbicula *spp. collected from the Western European river basins Meuse, Seine, Rhine and Rhône. In our phylogenetic dataset we included published *COI *haplotypes of European, African, American, Asian and Australian lineages. Moreover, we explored the presence of mismatches between mtDNA and nrDNA/morphotype (cytonuclear mismatches) in European populations, indicative of androgenesis between lineages and therefore challenging the sister-taxon relationships and the species status within the genus *Corbicula*. Furthermore, we emphasize the possible relationship between androgenesis and the invasive success of *Corbicula *clams.

## Results

### Morphological analysis

**Figure 1:**
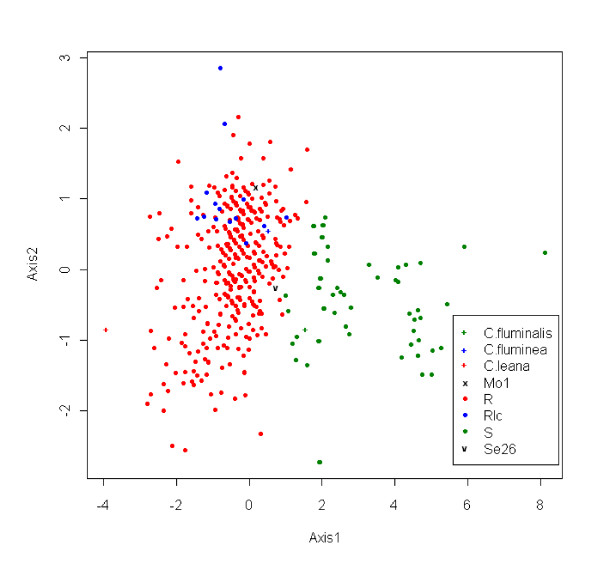
**Relationship between scores on Axis 1 and Axis 2 for the Principal Components Analysis for shell measurements on 429 specimens of *Corbicula***. R in red, Rlc in blue and S in green represent individuals of forms R, Rlc and S respectively. The type specimens of *C. fluminalis*, *C. fluminea *and *C. leana *are represented by symbols. Mo1 and Se26 are the two individuals exhibiting a mitochondrial-morphotype mismatch.

Two main morphotypes have been discriminated visually. They correspond to the forms described by Pfenninger *et al*. [42]: a round and broad form with deep ridges (round form, R) and a narrow form with closely spaced ridges (saddle form, S) (see [39] for pictures of both forms). Both forms occur syntopically in the Hollands Diep (Rhine-Meuse delta), in the River Meuse (Belgium, Table 3) and in the Rivers Seine and Rhine (Table 4). The individuals from the River Rhône can be subdivided into two colour groups of form R [36], viz. one with dark shells whose interior surface is white and purple (as in form R sampled elsewhere), the other with lighter shells whose interior is white-yellow (light form, Rlc). Form Rlc from the Rhône has also been recorded in the River Gardon (France, see [39]). Measurements were made on 429 specimens of *Corbicula *from all sampled localities. The measurements provided in the literature for the lectotypes of *C. fluminea *Müller and *C. fluminalis *Müller (collection of Universitetets Zoologisk Museum of Copenhagen [20]) and the type specimen of *C. leana *Prime (original description of the species in [26]) were also included in the analysis. The two main forms identified *a priori *(R and S) could be distinguished on the basis of the Principal Components Analysis (PCA) conducted on the three studied ratios (H/L, H/W and L/W, see Methods) (Figure 1). Form Rlc from the Rhône was morphometrically close to form R (Figure 1). We therefore conducted a new PCA on these two forms exclusively (results not shown). This analysis still showed an overlap between the two forms but suggested that specimens of form Rlc have a significantly lower H/L ratio (analysis of variance, p < 0.01). However, our study included only 14 specimens of form Rlc and a more extensive sampling is needed to test the differences with form R. The morphological distinction between forms R and Rlc is mainly based on colour, especially of the inner shell. Moreover, we noticed that juvenile individuals of form Rlc have an orange spot on their umbo (the dorsal shell protrusion) which is not observed for juveniles of form R (Pigneur LM & Marescaux J, personal observation).

**Table 3:** Sampling sites of *Corbicula *spp. in River Meuse.

Origin	Location (collectors)	Form (R, Rlc, S)	N *COI*
**Meuse**	Revin	R	5
(France)	Vireux-Molhain	R	7
	Chooz	R	2
**Meuse**	Heer-Agimont	R	7
(Belgium)	Hastière	R	10
	Waulsort	R	12
	Dinant	R	7
	Houx	R	8
	Godinne	R	6
	Rivière	R	4
	Tailfer	R	8
	Beez *(provided by divers)*	R	3
	Sclayn	R	5
	Huy	R	6
	Tihange (nuclear power plant)	R and S	17
	*(provided by C. Lamine - C. Martin)*		
	Amay	R and S	22
	Liège-Monsin	R	1
	Hermalle-sous-Argenteau	R	5
	Lixhe	R	7
**Meuse**	Cuijk	R	8
(Netherlands)	Alem	R	8
**Hollands Diep**	Moerdijk	R and S	7
(Rhine-Meuse delta,	*(provided by M. Greijdanus - B. Reeze)*		
Netherlands)	'Midden' *(provided by B. Reeze)*	R and S	11

The sites are presented from upstream to downstream. Collector names are indicated when different from the authors. N *COI *= number of individuals sequenced for *COI*.

**Table 4:** Details of the populations of *Corbicula *studied.

River	Country	Location	Form (R, Rlc, S)	Collectors	N *COI */*cyt b*
Meuse	France	See Table3	R	See Table3	176/20 (Table 3)
	Belgium		R, S		
	Netherlands		R, S		

Seine	France	Poses	R	Authors	6/3
			S		6/3

Rhine	Germany	Köln	R	M. Weitere - C. Viergutz	10/5
			S		3/3

Rhône	France	Creys	R	J. Mouthon	5/2
			Rlc		9/5

Origin, collector and number of individuals sequenced for *COI *and *cyt b *genes. (R = form R, S = form S, Rlc = form Rlc, N *COI */*cyt b *= number of individuals sequenced for *COI */*cyt b*).

The lectotype of *C. fluminea *clustered with individuals of morphotypes R and Rlc on the biplot. The type specimen of *C. leana *was found in the margin of the group composed of individuals of form R only. The lectotype of *C. fluminalis *was found within the group of specimens of form S (Figure 1). The two individuals (Mo1 and Se26) showing a cytonuclear mismatch (see *COI and cyt b sequences analysis *below) were morphologically part of the R-Rlc group (Figure 1).

### Sperm morphology

The sperm morphology of the three morphotypes of *Corbicula *(forms R, S and Rlc) was investigated because the biflagellate sperm type has been recognized as a diagnostic marker of androgenetic reproduction in this genus [15,16,18]. The analysis revealed biflagellate spermatozoa in the three morphotypes of *Corbicula *(forms R, S and Rlc). These three forms thus seem to reproduce through androgenesis like the invasive lineages found in America [18].

### *COI *and *cyt b *sequences analysis

*COI *and *cyt b *sequences were obtained respectively from 215 and 41 specimens from Meuse, Seine, Rhine and Rhône (Tables 3, 4). The analysis revealed only three haplotypes each corresponding to one of the three morphotypes. All individuals of a same morphotype exhibited exactly the same haplotype (except for 2 individuals, see below). Sequence comparisons between the three *COI *haplotypes revealed 22 variable sites distributed throughout the sequenced fragment (681 bp) (Alignment provided in Additional file 1). For *cyt b*, only 10 sites out of 326 were variable (Additional file 2). Since our *cyt b *sequences were relatively short (326 bp) and a lower number of sequences for this gene are available for *Corbicula*, we have conducted the sequence analysis and the phylogenetic study with *COI *only.

One haplotype (*COI *haplotype 1) was found in all our sampled populations. This haplotype 1 was exclusively found in form R and its nucleotide sequence is identical to European *C. fluminea *(haplotype I, [40]) and haplotype H2 [41], the American form A [18,34], the Japanese *C. leana *[34] and the Asian haplotype FW5 [31] (Tables 1, 2). We will refer to this *COI *haplotype as haplotype FW5 which was a common code used in Park & Kim [31] for all individuals of *Corbicula *with this *COI *sequence.

The second haplotype (*COI *haplotype 2) was found in all specimens of form S and in two specimens of form R (Table 2). Haplotype 2 is identical to the European "*C. fluminea*-like" *COI *haplotype IV from the Rhône [40] and haplotype H4 found in form S in the Rhine [42] and to the American form C [18] (Tables 1, 2). We will refer to this haplotype as haplotype FW17 as in Park & Kim [31]. Haplotype FW17 has not been found in any Asian population [31].

We observed a discrepancy between the mitochondrial lineage and morphology for two individuals of form R. The first one was a single individual from the River Meuse at Liège-Monsin (individual Mo1) and the second one is a single individual from the Seine (individual Se26). Both individuals had the morphotype R but exhibited the *COI *haplotype 2 (found in form S). The same results were obtained for *cyt b *haplotypes. We sequenced both genes again on new tissue samples from these two individuals and obtained the same results.

**Figure 2:**
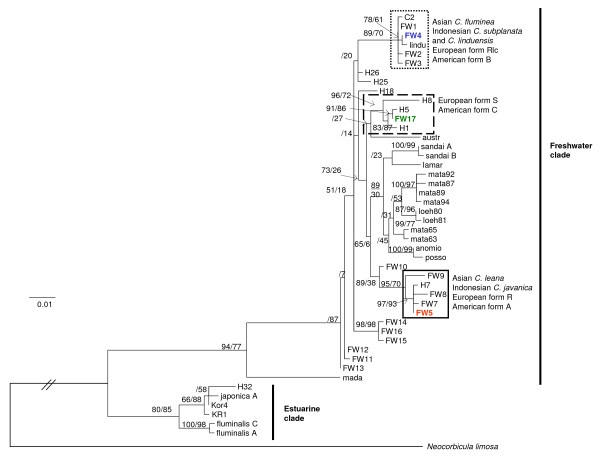
**Maximum Likelihood tree based on a 562 bp fragment of the mitochondrial *COI *gene from *Corbicula *spp**. Posterior probabilities from Bayesian Inference (first) and bootstrap values from Maximum Likelihood (second) are indicated for nodes found in both analyses. Sequence of *Neocorbicula limosa *was used as an outgroup. See Table 1 for the origin of all the sequences. The three European morphotypes are indicated (form R, form S and form Rlc) and the frames are colour coded according to Fig. 1. Two individuals of form R exhibited the *COI *haplotype of form S (haplotype 2 = FW17).

Our third haplotype (*COI *haplotype 3) was exclusively found in the individuals of form Rlc from the Rhône while form R from this same site had the haplotype 1 (FW5) (Table 2). Renard *et al*. [40] found *COI *haplotype IV in the five individuals studied from the Rhône; we did not find this haplotype IV in the new samples we obtained from River Rhône. Our haplotype 3 from the Rhône is identical to the sequence of *C. fluminalis *(haplotype V) from Renard *et al*. [40] (Table 2). Therefore, there is a discordance between our haplotypes and those obtained by Renard *et al*. [40] for form S. The same discrepancy was also reported by Pfenninger *et al*. [42] (Table 2). Our haplotype 3 is identical to the haplotype FW4 of the Indonesian *C. subplanata *[31,50] (Table 1). We will here refer to haplotype FW4 as in Park & Kim [31]. This haplotype FW4 clusters with the American haplotype B1 (identities: 636/637 bp) and *C. fluminea *from Korea (identities: 613/614 bp) (Figure 2).

Our analysis yielded only three haplotypes for all studied individuals among the four sampled European rivers. Surprisingly, Pfenninger *et al*. [42] recorded a larger number of *COI *haplotypes, especially in the River Rhine. We aligned all of these haplotypes and observed that some sequences contain several Ns or are shorter than most published sequences. We therefore included only some representative haplotypes of that study in our Maximum Likelihood (ML) and Bayesian Inference (BI) analyses.

### Mitochondrial phylogeny (*COI *gene)

In total, 57 COI sequences of *Corbicula *available in GenBank, including those three sequences from the present study, were included in the phylogenetic analysis (Table 1). Out of these 57 sequences, 47 *COI *haplotypes were defined (Additional file 1). From the 714 positions (562 excluding gaps or missing data) including the outgroup, 158 sites were variable (28%). Only 13% of the sites remained variable when we excluded the outgroup, the estuarine lineages (see Figure 2) and *C. madagascariensis*.

Based on the lower value of Akaike information criterion (AIC), the GTR+γ model was selected for phylogenetic analysis. *Neocorbicula limosa *was used as an outgroup. ML analysis and BI gave a similar topology. The phylogenetic tree (Figure 2) shows two divergent clades: an "estuarine clade" (Asian *C. fluminalis *and *C. japonica*) and a "freshwater clade". Within this freshwater clade, *C. madagascariensis *is a sister-taxon to all other freshwater lineages of *Corbicula*. In this latter group, there is no clear geographic clustering of haplotypes of *Corbicula*, highlighting their cosmopolitan distribution. However the phylogenetic relationships between the freshwater lineages of *Corbicula *remain unresolved due to the low support (bootstrap and posterior probabilities) obtained for most clades. The three haplotypes of our study (referred to as FW4, FW5 and FW17 as in Park & Kim [31]) are each associated with a distinct freshwater subclade. Within each of these subclades, there are mostly representatives of *Corbicula *with biflagellate sperm (see Table 1) suggesting that these lineages reproduce through androgenesis [19]. However the clade containing haplotype FW4 also includes *C. linduensis *which has monoflagellate sperm [17], suggesting that this latter reproduces sexually.

### Microsatellites

Nuclear markers were mainly used in this study to investigate the observed mismatches between mtDNA and nrDNA/morphotype. For all eleven polymorphic micosatellite loci, the two individuals with morphotype R and *COI *haplotype 2 (FW17) of form S (individuals Mo1 and Se26) exhibited exactly the same microsatellite genotype as the form R individuals. Therefore, both individuals clearly belong to the form R lineage at the morphological and nuclear levels but have the form S mitochondrial haplotype.

The same eleven microsatellite loci were also used to analyse several West European individuals of *Corbicula *belonging to the three different morphotypes (Table 5). For microsatellite locus ClB03, forms R and S present the same alleles (heterozygous; 2 alleles) while for the loci ClA02, ClB11 and C01 forms S and Rlc have the same allele size. Two loci (ClA01 and ClA03) did not amplify in form S, though they did amplify in the two specimens of form R with mtDNA *COI *haplotype 2 (as in form S). Two other loci (ClD06 and ClE01) did not amplify in form Rlc. The remaining four microsatellite loci amplified in all three forms and revealed differences in allele size between form R, S and Rlc. Since the microsatellite library was constructed for form R [51], it is not surprising that, due to the genetic divergence between the three forms, some loci did not cross-amplify in form S or Rlc (Table 5).

**Table 5:** Characteristics of polymorphic microsatellite loci and amplification in the three morphotypes of *Corbicula *of Western Europe.

Primer name	Morphotype	Repeat type	Ta (°C)	N	Size (bp)
**ClA01**		(gt)			
	**R**		53	27	198
	**S/R**		53	2	198
	**S**		53	23	-
	**Rlc**		53	15	196

**ClA02**		(tg)			
	**R**		53	27	110-114
	**S/R**		53	2	110-114
	**S**		53	23	112-116
	**Rlc**		53	15	112-116

**ClA03**		(tg)			
	**R**		53	27	192-194
	**S/R**		53	2	192-194
	**S**		53	23	-
	**Rlc**		53	15	190

**ClB03**		(ag) (ga)			
	**R**		53	27	233-239
	**S/R**		53	2	233-239
	**S**		53	23	233-239
	**Rlc**		53	15	239

**ClB11**		(ca)			
	**R**		53	27	311
	**S/R**		53	2	311
	**S**		53	23	311-313
	**Rlc**		53	15	311-313

**ClC01**		(gt)			
	**R**		53	27	175-179
	**S/R**		53	2	175-179
	**S**		53	23	173-175
	**Rlc**		53	15	173-175

**ClC08**		(ct) (ct)			
	**R**		55	27	216-220
	**S/R**		55	2	216-220
	**S**		55	23	260-306-(316)
	**Rlc**		55	15	306

**ClC12**		(ac)			
	**R**		53	27	226
	**S/R**		53	2	226
	**S**		53	23	226-228
	**Rlc**		53	15	230

**ClD06**		(gcgt)			
	**R**		53	27	199-207
	**S/R**		53	2	199-207
	**S**		53	23	237
	**Rlc**		53	15	-

**ClD12**		(gtgc)			
	**R**		53	27	274-278
	**S/R**		53	2	274-278
	**S**		53	23	264-274
	**Rlc**		53	15	274

**ClE01**		(tg)			
	**R**		53	27	213
	**S/R**		53	2	213
	**S**		53	23	209
	**Rlc**		53	15	-

Ta = annealing temperature, N = number of individuals genotyped, Size = allele(s) size(s). S/R represents the two individuals with the *COI *haplotype 2 (like in form S) and the morphotype R. "R", "S" and "Rlc" refer to the three West European morphotypes.

Surprisingly, for each tested microsatellite locus, zero polymorphism was observed between individuals within each morphotype originating from different West-European populations. Only three distinct genotypes (genotypes 1, 2 and 3 for forms R, S and Rlc respectively) were identified. Bayesian clustering conducted using the STRUCTURE software revealed distinct categories, with the highest and most reliable probabilities for K = 3 (Additional file 3). This confirmed the observed differences between the three forms and the presence of only three invasive haplotypes/genotypes of *Corbicula *in Western Europe (Table 5).

These results are indicative of an overall very low genetic divergence within European *Corbicula *and could be related to their clonal reproductive mode.

## Discussion

### - Three European morphotypes/haplotypes/genotypes

The dark-coloured form R individuals we found correspond morphologically to the description of *C. fluminea *in [36,41,42]. Our morphological analysis did not provide a strong taxonomic assignment although the type specimen of *C. leana *was plotted near the group including form R. The sequence of haplotype 1, found in all studied individuals of form R in Europe (except the two "mismatches"), was identical to Asian haplotype FW5. Those results are congruent with those of Siripattrawan *et al*. [34] and Hedtke *et al*. [19] which concluded that populations of American form A (and therefore the European form R) are derived from populations of *C. leana *(haplotype FW5) from Japan. On the basis of these *COI *results, the European form R would not correspond to *C. fluminea *sensu stricto as previously assumed. Regarding the genotyping results, all 27 individuals of form R had one specific nuclear genotype (genotype 1) for the 11 tested loci.

The individuals of form S (Meuse, Seine and Rhine rivers) correspond morphologically to *Corbicula fluminalis *as defined by Renard *et al*. [40] and Korniushin [41]. Our morphological analysis, through the PCA, also showed similarity between the European form S and the lectotype of *C. fluminalis *from Mesopotamia. The *COI *haplotype 2 found in form S was identical to haplotype FW17 and to American haplotype C1 (of form C). Interestingly Park & Kim [31] already highlighted that no Asian haplotype corresponds to haplotype FW17. Genetic and morphological data of specimens from the native area of *C. fluminalis *(Middle East, Caucasus and Central Asia) are needed to confirm the taxonomic assignment of the European form S/American form C. Our results also suggest that form S does not correspond to *C. fluminalis *sensu Park & Kim [31]. This Chinese, estuarine *C. fluminalis *was found in the same mitochondrial clade as *C. japonica *(Figure 2). Korniushin [41] already reconsidered this form as *C. cf. japonica *on the basis of anatomy and reproductive features. The genotyping results showed that all 23 European individuals of *Corbicula *of form S had the same, distinct nuclear genotype (genotype 2) for the 9 microsatellite loci that amplified in form S although these nuclear markers were developed for form R.

We obtained new samples from the Rhône and our analysis revealed the presence of two distinct *COI *lineages in this river: (i) the haplotype 1 (FW5) in individuals of form R and (ii) the haplotype 3 (FW4) found in specimens of form Rlc exclusively. The European light form Rlc had the same *COI *haplotype as the Indonesian *C. subplanata*, and was closely related to the American form B and *Corbicula fluminea *from Asia [19,34]. Glaubrecht *et al*. [10,17] already made the assumption that *C. subplanata *would be conspecific with the widespread *C. fluminea*. Hedtke *et al*. [19] concluded that the American form B would be derived from populations of the Asian lineage *C. fluminea*. As in form S, 9 microsatellite loci amplified in European form Rlc and revealed a third distinct genotype (genotype 3), being identical in all tested individuals of form Rlc.

The overall low genetic divergence between and within the three forms and the possibility of a peculiar hybridization through androgenesis between forms (see below) suggest that invasive lineages of *Corbicula *may represent a polymorphic species complex. Therefore we propose to use the nomenclature "form R", "form S" and "form Rlc" for the three West-European lineages of *Corbicula*.

### - Unravelling discordant COI results

The sequence of haplotype 3 (FW4) we found in form Rlc (Rhône) was identical to the *C. fluminalis *- haplotype V and not to the expected haplotype IV of the Rhône found by Renard *et al*. [40]. This result of Renard *et al*. [40] is contrasting with our data and those of Pfenninger *et al*. [42]. Moreover, Renard *et al*. [40] included 6 individuals of the *C. fluminalis *form (form S) from the Meuse (Netherlands) but their sequence (*COI *haplotype V) does not correspond to the one we found (haplotype 2) in our sampling of morphotype S and it does not correspond to haplotype H4 from form S published by Pfenninger *et al*. [42] (see Table 2). We do not have any evidence of temporal changes in the studied populations and we therefore hypothesize a mismatch between the published sequences and their associated names/populations in the paper of Renard *et al*. [40]. More specifically, the *COI *sequence assigned to *C. fluminalis *(haplotype V) in their publication actually belongs to the individuals from the Rhône (their third unidentified lineage) and corresponds to our form Rlc (also from the Rhône), while their sequence of haplotype IV (Rhône) corresponds to their specimens of *C. fluminalis *as Pfenninger *et al*. [42] and this study demonstrated. In Table 2, we present a summary of the incongruence and a comparison of European, American and Asian *COI *haplotypes of *Corbicula*.

### - Mitochondrial/morphotype-nuclear mismatch

In Europe and North America, most individuals of *Corbicula *with the same morphotype belong to the same mitochondrial lineage ([18,19,42], this study). In addition, in this study we have preliminary indications that the same *Corbicula *morphotype also belongs to a single genotype although more individuals should be investigated. However, we detected a discrepancy between the mitochondrial lineage and, the morphology and the nuclear lineage of two specimens of form R, one from the Meuse and one from the Seine, two of the three rivers where we found forms R and S in sympatry. These individuals with morphotype R exhibit the mitochondrial haplotype of form S (for both *cyt b *and *COI*) but all polymorphic microsatellite markers identified a form R nuclear DNA origin (genotype 1) for both specimens. In the absence of recent nuclear exchange between the morphotypes, the observed mismatch could be explained by double uniparental inheritance (DUI), population polymorphism in the ancestor of the species or egg parasitism between the species [19]. Hedtke *et al*. [19] did not find any evidence of DUI in individuals presenting a mtDNA/morphotype mismatch. The hypothesis of a polymorphic ancestor was rejected on the basis of their phylogenetic analysis [19]. Hedtke *et al*. [19] therefore concluded that this observed discrepancy could only be the consequence of an androgenetic "egg capture": after fertilization between the unreduced sperm of one haplotype and the egg of a distinct haplotype, the maternal nuclear DNA from the egg is extruded. The descendant therefore contain paternal nuclear DNA while they retain the maternal mitochondrial DNA, resulting in a cytonuclear mismatch as observed here for two individuals.

A similar cytonuclear mismatch was observed in some American populations between forms A and B and between forms B and C living in sympatry [18,19]. Discrepancies between nuclear and mitochondrial phylogenies have already been reported for *Corbicula *[42,44]. According to our mitochondrial phylogeny and our microsatellite data, the disjunction found in our two singular individuals would imply a spermatozoon of lineage R that "parasitized" an egg of lineage S.

### - Androgenesis and invasiveness

In freshwater ecosystems, *Corbicula fluminea *sensu lato is considered one of the most invasive species [3] because of its large geographic dispersal and invasive behaviour [3,20,24]. The invasive success of lineages of *Corbicula *is attributed to their following biological traits: rapid growth and maturation, high fecundity, excellent dispersal capacities and association with human activities [3]. However, their unique reproductive mode might also play a role in the invasiveness of lineages of *Corbicula *while this has never been highlighted. Our phylogenetic analysis (Figure 2) demonstrates that the "freshwater clade" includes both sexual dioecious and androgenetic lineages. The sexual lineages are mostly restricted to limited Asian areas, e.g. *C. sandai *which is endemic to Lake Biwa (Japan) or *C. matannensis *which is endemic to Indonesia. Moreover, it has been shown that, on a limited spatial scale, the lacustrine lineages of *Corbicula *from Sulawesi (Indonesia) form well-defined genetic clades [50] while identical or closely related haplotypes of the androgenetic lineages are found over large geographic distances, both in the native and invaded regions ([31,50], this study). This emphasizes that androgenesis seems to favour the invasive success of these clams. More specifically, androgenetic *Corbicula *are hermaphrodites and capable of self-fertilization. Therefore, a single individual may easily found a new population if the conditions are favourable. This is one of the main advantages of asexual as compared to sexual lineages [52].

In addition, our genetic analysis of European populations of *Corbicula *revealed an absence of polymorphism within each form. Many invasive species escape low levels of genetic diversity owing to multiple and/or repeated introduction events [53,54]. On the contrary, there is a "prevalence of genetic bottlenecks" in invasive species with several species showing a low genetic diversity but a high invasive potential [55]. This is considered the "genetic paradox of invasive species" [55-58]. In the case of *Corbicula*, further investigations should assess the genetic diversity of the native populations to determine whether the invasive forms have encountered genetic bottlenecks or not. Due to androgenesis several of the lineages in the native area may indeed contain a low genetic diversity. Whatever the origin of their low genetic diversity, invasive lineages of *Corbicula *represent one of those examples of "genetic paradox". Phenotypic plasticity, which is particularly high in molluscs, might have played an important role in the adaptation to and colonization of new environments as demonstrated in many other invasive species [59-61]. In other words, those three invasive clonal lineages of *Corbicula*, particularly form R dominating in Western Europe, could be "general purpose genotypes" or even "super-genotypes" associated with high levels of plasticity in response to fluctuating environmental conditions [62,63].

However, a certain level of genetic diversity in *Corbicula *may be found in the combination of a nuclear genome of one lineage with a new mitochondrial genome of a different lineage as a result of androgenesis between lineages. This may provide a higher advantage to androgenetic lineages compared to other asexually reproducing species. Mitochondrial capture has already been pointed out as dispersal strategy in the endangered cypress tree *Cupressus dupreziana*. This androgenetic organism produces diploid pollen that may in turn fertilize the ovule of the related, common species *Cupressus sempervirens*. This latter species, then, acts as a surrogate mother [64,65]. Moreover, androgenesis allows an elevation of ploidy when there is mixing of nuclear genomes during incomplete extrusion of the maternal nuclear genome [19,45,46].

### - Androgenetic species: the pitfall of mitochondrial phylogenies

Several studies revealed mitochondrial/morphotype discrepancies in populations of *Corbicula *where distinct forms live in sympatry ([18,19,42], this study) and hypothesized that it was the consequence of an androgenetic egg parasitism ([18,19], this study). We would like to highlight here that this type of mitochondrial capture could lead to inaccurate species delimitation. The mitochondrial gene tree only traces the maternal lineage and it is, in the case of androgenesis, misleading for species relationships. Until now, most phylogenetic studies in *Corbicula *have relied mainly or solely on mitochondrial data, but this can result in the grouping of distinct nuclear lineages in the same mitochondrial cluster. Therefore the use of an "integrative taxonomy" approach as developped here, combining nuclear and mitochondrial data along with morphology, is essential to verify the phylogenetic relationships within androgenetic taxa such as *Corbicula*. Moreover, we suggest that due to these peculiar hybridization events between lineages as a consequence of an androgenetic mode of reproduction, invasive European *Corbicula *should be considered as a polymorphic species complex including three distinct clusters. For example, the European form S and the American form C have the same *COI *sequence but appear very different on the basis of shell morphology compared to pictures of form C from Lee *et al*. [18]. The observed differences in shell shapes could be related to phenotypic plasticity, but alternatively, forms S and C could also belong to distinct nuclear lineages sharing the same mitochondrial DNA through androgenesis. The same hypothesis seems confirmed by preliminary results in the *COI *cluster "form B - form Rlc". These morphs can easily be distinguished: the form B from the Americas has a deep purple inner shell surface while the form Rlc from Europe has a whitish inner shell surface. The assessment of cross-amplification of microsatellite loci in American forms revealed that form B and form Rlc have distinct genotypes [51]. They therefore belong to distinct clusters at the nuclear level.

## Conclusions

The three different morphotypes of *Corbicula *found in Europe are clearly distinct at the mitochondrial and nuclear level. The European form S seems related to *C. fluminalis *on the basis of morphology while mitochondrial data suggest that forms R and Rlc are derived from the androgenetic *C. leana *and *C. fluminea *respectively. However this taxonomic assignment remains uncertain and, due to the weak phylogenetic signal among freshwater *Corbicula*, these could be considered as a polymorphic species complex. Moreover, species delineation in *Corbicula *is even more complicated since we have to take into account the possibility of androgenesis and egg capture between the different morphotypes as observed in this study for two individuals. Hedtke *et al*. [19] also demonstrated that nuclear exchange is possible and that the nuclear DNA of the American *Corbicula *form B originated from hybridization of separate nuclear lineages. As a consequence, taxonomic assignment in androgenetic lineages based on mitochondrial phylogeny only may be biased, and using an "integrative taxonomy" approach combining nuclear and mitochondrial data along with morphology may at least avoid the pitfall of pooling divergent evolutionary nuclear lineages. We therefore recommend using the proposed nomenclature form R, form S and form Rlc for convenience in differentiating the three invasive European *Corbicula *lineages.

Furthermore our phylogeny highlights that the cosmopolitan, invasive forms of *Corbicula *have identical or closely related haplotypes and seem to be androgenetic while the sexual, dioecious lineages seem restricted to the native Asian areas. Androgenesis would thus play an important role in the invasive success of *Corbicula *clams, especially because they are hermaphrodites and capable of self-fertilization.

## Methods

### Specimen collection

Live specimens were collected from 23 localities in the Meuse river in France, Belgium and the Netherlands (Table 3), and from one site in the Seine river in France (Table 4). Additional specimens from one locality in the Rhine and one in the Rhône were obtained (Table 4). Specimens were immediately processed or preserved in 96% ethanol prior to analysis. Individuals from the Meuse and Seine rivers were collected in fish bypasses with a handnet or directly from the river bottom with a handnet, or dug out from the sediment with an Eckman dredge.

### Morphological analysis

The shell morphology of the different morphs from Meuse, Rhine, Rhône and Seine rivers was analysed. The shells of 429 individuals were measured for length (L), height (H) and width (W) with a calliper: "L" is the maximal anteroposterior dimension, "H" is the maximal dorsoventral dimension and "W" is the maximal width of the valves. We also included published measurements of the lectotypes of *C. fluminea *and *C. fluminalis *[20] and of the original description of *C. leana *[26]. A Principal Component Analysis (PCA) was carried out using the table of the three morphometric ratios (H/L, H/W and L/W). Calculations were performed and graphics drawn using R software with the ade4 package [66]. The function "dudi.pca" was used to apply the PCA to the correlation matrix. Scores from axis 1 were plotted against those from axis 2, each point was identified individually.

### Sperm morphology

Biflagellate sperm is indicative of androgenesis in the genus *Corbicula *[15,16,18]. We analysed the sperm morphology of specimens of form R (Meuse, N = 4), form S (Rhine and Seine, N = 5) and form Rlc (Rhône, N = 3). To isolate and observe the spermatozoa, a protocol was adapted from several studies [67-69]. The individuals preserved in absolute ethanol were opened and the visceral mass, which includes the gonads, was cut. The tissue was collected in a tube and collagenase resuspended in RPMI was added (final concentration: 1 mg/l). The tissue was sheared and collagenase-RPMI mix was added to a final volume of 600 μl. The preparation was then incubated at 45°C until complete degradation. The samples were centrifuged during 3 minutes at 3000 rpm. One drop of the undernatant was put on a glass slide and observed under a light microscope at 100× magnification.

### Gene amplification and sequencing

Total genomic DNA was extracted from the adductor muscles or the foot of 215 individual specimens (fresh or preserved in 96% ethanol) using the DNeasy blood & tissue kit (Qiagen) according to the manufacturer's protocol. A fragment of 710 bp of the mitochondrial cytochrome *c *oxidase subunit I (*COI*) gene and a fragment (430 bp) of the cytochrome *b *(*cyt b*) gene were amplified by Polymerase Chain Reaction (PCR) using the primers LCOI490 and HCO2198 [70] and, UCYTB151F and UCYTB270R, respectively [71]. Amplifications were performed in 25 μl total volume including 0.5 μl of gDNA, 1× GoTaq Green reaction buffer (Promega), 200 μM of dNTPs (Promega), 0.5 μM of both primers and 0.1 U of GoTaq DNA polymerase (Promega). PCR conditions were: 4 min at 94°C followed by 30 cycles of 45 s at 94°C, 45 s at annealing temperature (45 °C for *COI *and 42°C for *cyt b*) and 45 s at 72°C, and then a final extension of 10 min at 72°C. PCR products were purified and sequenced with each universal primer on an automated ABI3730XL Genetic Analyzer (Macrogen Inc.). *COI *and *cyt b *sequences were deposited in GenBank [GU721082-GU721084; JF518976-JF518978]. Sequences were visualized and aligned using BioEdit 7.0.5.3 [72].

### Phylogenetic analyses (*COI*)

The evolutionary model for the phylogenetic analysis of *COI *sequences was selected using jModelTest 0.1 software [73]. Maximum-likelihood trees were constructed with PhyML 2.4.5 software and bootstrap values were obtained for 1,000 replicates [74]. A Bayesian Inference (BI) analysis was performed using MrBayes 3.1.2 [75]. We ran four independent analyses with four Markov Chain Monte Carlo (MCMC) each, for 4 million generations. Trees and parameters were sampled every 100 generations. To ensure stabilization, we discarded the first 25% of sampled trees. The remaining trees were used to compute a consensus tree. The split frequency was below 0.01. For all analyses, published *COI *sequence of *Neocorbicula limosa *was used as an outgroup. A total of 54 sequences from several *Corbicula *lineages available on GenBank were included (origins in Table 1). Trees were visualized using MEGA4 [76].

### Microsatellite study

We used eleven microsatellite loci described in Pigneur *et al*. [51] to analyze individuals of the three observed European morphotypes (see Table 5 for the number of tested individuals). Regarding the samples from the Meuse, Rhône and Rhine, the individuals analyzed in the present study are different from those used in Pigneur *et al*. [65]. For each locus, the amplification was performed in 10 μl total volume including 0.5 μl of gDNA, 1× GoTaq reaction buffer (Promega), 200 μM of dNTPs (Promega), 0.5 μM of both primers and 0.1 U of GoTaq DNA polymerase (Promega). The forward primers were 5' fluorescently labelled and fitted with a GTTTCTT PIG-tail. Amplification by PCR was performed as follows: 4 min at 94°C followed by 30 cycles: 45 s at 94°C, annealing for 45 s and 72°C for 45 s, and then a final extension of 12 min at 72°C. The fragments were analysed on an ABI 3130XL Genetic Analyzer with GeneScan-500 (LIZ) size standard (Applied Biosystems). Results were visualized using GENEMAPPER software (Applied Biosystems). Additionally, the microsatellite results were analyzed using Bayesian clustering with the STRUCTURE software version 2.0 [77] to define the number of categories (K) of individuals. STRUCTURE was run for 10 repetitions of each K from 1 to 5 assuming admixture. Analysis was performed using a burn-in period of 50,000 followed by 200,000 MCMC repetitions.

## Authors' contributions

LMP conceived the study, carried out all the experimental work, performed the phylogenetic and statistical analyses and drafted the manuscript. JM, KR and EE participated in the field sampling and the molecular genetic analyses. JPD participated in the design of the study and revised the manuscript. KVD conceived the study, participated in its design and coordination and helped to draft the manuscript. All authors read and approved the final manuscript.

## Supplementary Material

Additional file  1 **Alignment file of the 47 *COI *haplotypes of *Corbicula *spp. used in the present study**. Click here for file

Additional file  2 **Alignment file of the three *cyt b *sequences of the three European morphotypes of *Corbicula *(R, S and Rlc)**. Click here for file

Additional file  3 **Estimated population structure of *Corbicula *spp. for K = 3 and mean Ln P(D) ±SD for 10 replicates at each level of K clusters (from 1 to 5)**. Click here for file
