# Supplementary figures and images for: Phylogeny and androgenesis in the invasive Corbicula clams (Bivalvia, Corbiculidae) in Western Europe
Source: BMC Evol Biol. 2011 May 27;11:147. doi: 10.1186/1471-2148-11-147 (PMC3126740; doi:10.1186/1471-2148-11-147)

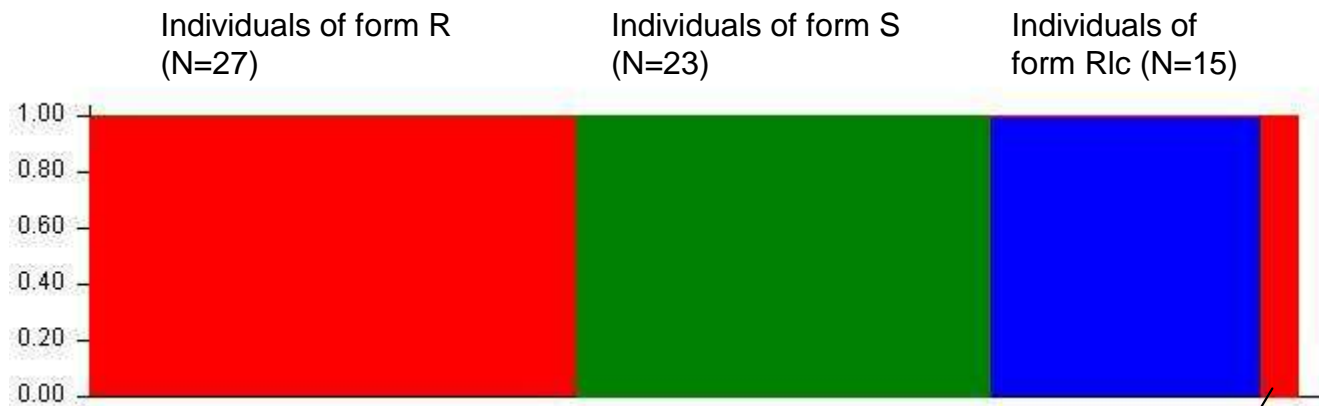

Individuals with mismatch between  
mtDNA (form S) and nrDNA (form R) (N=2)

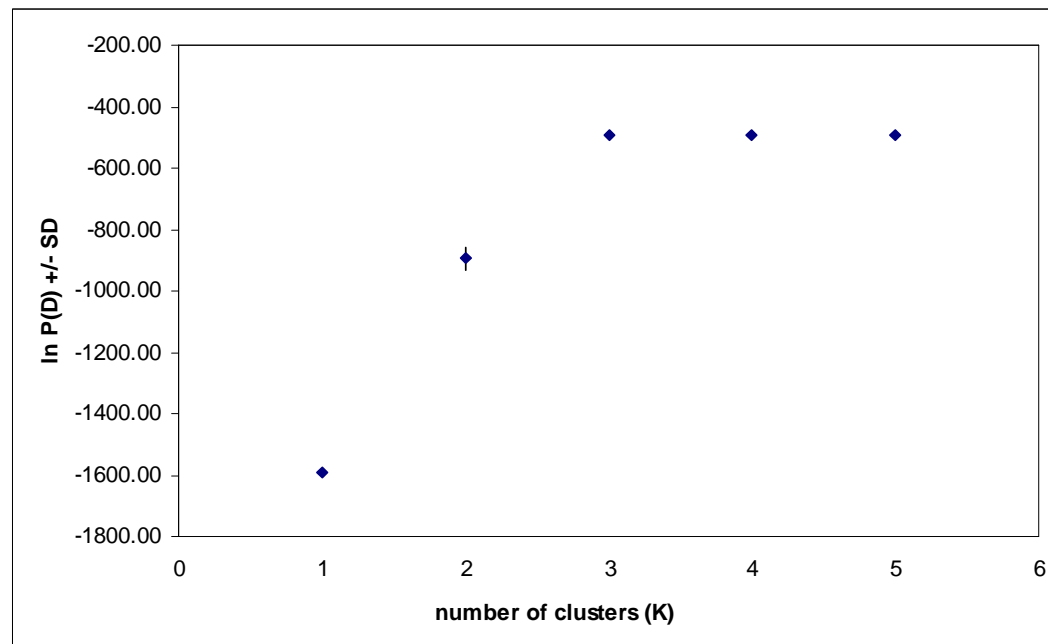

Supplement: Additional file 3 — Estimated population structure of Corbicula spp. for K = 3 and mean Ln P(D) ±SD for 10 replicates at each level of K clusters (from 1 to 5). [file 1471-2148-11-147-S3.PDF]
